# Supplementary material for: Human receptive endometrial assembloid for deciphering the implantation window
Source: eLife. 2026 Apr 1;12:RP90729. doi: 10.7554/eLife.90729 (PMC13043174; doi:10.7554/eLife.90729)
Supplement: Supplementary file 1. — Table A Composition of expansion medium (ExM) of endometrial assembloid. Table B Composition of hormone regimen of endometrial assembloid. Table C Composition of modified In Vitro Culture Medium (mIVC1 and mIVC2) for co-culture of blastoids and endometrial assembloids. [file elife-90729-supp1.docx]

**Supplementary File 1 Composition of medium**

**Table A. Composition of expansion medium (ExM) of endometrial assembloid**

| Reagent | Manufacturer | Catalog no. | Concentrations |
| --- | --- | --- | --- |
| DMEM/F12 | Gibco | 11039-021 |  |
| Antibiotic-Antimycotic (100X) | Gibco | 15240062 | 1% |
| ITS | Gibco | 41400-045 | 1% |
| L-Glutamine | Gibco | 25030-081 | 2 mM |
| Nicotinamide | Sigma | N3376 | 1 mM |
| B27 | Gibco | 17504-044 | 2% |
| N2 | Gibco | 17502-048 | 1% |
| Noggin | Proteintech | HZ-1118 | 100 ng/ml |
| EGF | Peprotech | AF-100-15 | 50 ng/ml |
| FGF2 | Origene | TP750002 | 100 ng/ml |
| WNT-3A | Proteintech | HZ-1296 | 200 ng/ml |
| R-Spondin-1 | Peprotech | 120-38 | 200 ng/ml |
| A83-01 | MCE | HY-10432 | 0.5 uM |
| N-acetyl-L-cysteine | Sigma | A7250 | 1.25 mM |
| p38 inhibitor SB202190 | Sigma | SB202190 | 10 uM |

**Table B. Composition of hormone regimen of endometrial assembloid**

| Reagent | Manufacturer | Catalog no. | Concentrations |
| --- | --- | --- | --- |
| Estradiol | Sigma | E2758 | 10nM |
| Medroxyprogesterone Acetate | Selleck | S2567 | 1μM |
| N6,2′-O-dibutyryladenosine 3′,5′-cyclic monophosphate sodium salt (cAMP) | Sigma | D0627 | 1μM |
| Human Chorionic Gonadotropin (HCG) | Livzon Pharmaceutical Group Inc | 2000U | 1μg/ml |
| Human Placental Lactogen (HPL) | R&D Systems | 5757-PL | 20ng/ml |
| Prolactin | Peprotech | 100-07 | 20ng/ml |

**Table C. Composition of modified In Vitro Culture Medium (mIVC1 and mIVC2) for** **co-culture of blastoids and endometrial assembloids**

| Reagent | Manufacturer | Catalog no. | Concentrations |
| --- | --- | --- | --- |
| mIVC1 | | | |
| advanced DMEM/F12 | Gibco | 12634-010 | - |
| defined fetal bovine serum | Biosera | bs-0003 | 20% |
| L-glutamine | Gibco | 25030 | 2 mM |
| ITS-X | Gibco | 51500-056 | 1x |
| β-estradiol | Sigma | E8875 | 8 nM |
| progesterone | Sigma | P0130 | 200 ng/ml |
| N-acetyl-L-cysteine | Sigma | A7250 | 25 μM |
| sodium lactate | Sigma | L7900 | 0.22% |
| Sodium pyruvate | Sigma | P4562 | 1 mM |
| Y27632 | Selleck | S1049 | 10μM |
| mIVC2 | | | |
| advanced DMEM/F12 | Gibco | 12634-010 | - |
| KOSR | Gibco | A3181501 | 30% |
| L-glutamine | Gibco | 25030 | 2 mM |
| ITS-X | Gibco | 51500-056 | 1x |
| β-estradiol | Sigma | E8875 | 8 nM |
| progesterone | Sigma | P0130 | 200 ng/ml |
| N-acetyl-L-cysteine | Sigma | A7250 | 25 μM |
| sodium lactate | Sigma | L7900 | 0.22% |
| Sodium pyruvate | Sigma | P4562 | 1 mM |
| Y27632 | Selleck | S1049 | 10μM |
